# State-building and human resources for health in fragile and conflict-affected states: exploring the linkages

**DOI:** 10.1186/s12960-015-0023-5

**Published:** 2015-05-15

**Authors:** Sophie Witter, Jean-Benoit Falisse, Maria Paola Bertone, Alvaro Alonso-Garbayo, João S Martins, Ahmad Shah Salehi, Enrico Pavignani, Tim Martineau

**Affiliations:** ReBUILD Programme, Institute for International Health and Development, Queen Margaret University, Edinburgh, UK; Department of International Development & St Antony’s College, University of Oxford, Oxford, UK; Department of Global Health and Development, London School of Hygiene and Tropical Medicine & ReBUILD Programme, London, UK; Liverpool School of Tropical Medicine, Liverpool, UK; Faculdade de Medicina e Ciências da Saúde, Universidade Nacional Timor Lorosa’e, Dili, Timor-Leste; Department of Global Health and Development, London School of Hygiene and Tropical Medicine (LSHTM) & Health Economics and Financing Directorate, Ministry of Public Health, Kabul, Afghanistan; The School of Population Health, University of Queensland, Brisbane, Australia

**Keywords:** State-building, Human resources for health, Fragile states, Conflict-affected, Afghanistan, Timor-Leste, Burundi

## Abstract

**Background:**

Human resources for health are self-evidently critical to running a health service and system. There is, however, a wider set of social issues which is more rarely considered. One area which is hinted at in literature, particularly on fragile and conflict-affected states, but rarely examined in detail, is the contribution which health staff may or do play in relation to the wider state-building processes. This article aims to explore that relationship, developing a conceptual framework to understand what linkages might exist and looking for empirical evidence in the literature to support, refute or adapt those linkages.

**Methods:**

An open call for contributions to the article was launched through an online community. The group then developed a conceptual framework and explored a variety of literatures (political, economic, historical, public administration, conflict and health-related) to find theoretical and empirical evidence related to the linkages outlined in the framework. Three country case reports were also developed for Afghanistan, Burundi and Timor-Leste, using secondary sources and the knowledge of the group.

**Findings:**

We find that the empirical evidence for most of the linkages is not strong, which is not surprising, given the complexity of the relationships. Nevertheless, some of the posited relationships are plausible, especially between development of health cadres and a strengthened public administration, which in the long run underlies a number of state-building features. The reintegration of factional health staff post-conflict is also plausibly linked to reconciliation and peace-building. The role of medical staff as part of national elites may also be important.

**Conclusions:**

The concept of state-building itself is highly contested, with a rich vein of scepticism about the wisdom or feasibility of this as an external project. While recognizing the inherently political nature of these processes, systems and sub-systems, it remains the case that state-building does occur over time, driven by a combination of internal and external forces and that understanding the role played in it by the health system and health staff, particularly after conflicts and in fragile settings, is an area worth further investigation. This review and framework contribute to that debate.

## Introduction

Human resources for health are self-evidently critical to running a health service and system, and there has been a small but growing literature on the returns to investing in human resources for health (HRH) from the point of view of health and associated economics gains [1]. There is, however, a wider set of social issues which is more rarely considered. Health systems have a social function, and the individuals and institutions which make them up play roles beyond preventing and curing ill health and working to promote good health [2]. One area which is hinted at in literature, particularly on fragile and conflict-affected states, but rarely examined in detail, is the contribution which health staff may or do play in relation to the wider state-building processes.

This article aims to explore that relationship, developing a conceptual framework to understand what linkages might exist and looking for empirical evidence in the literature to support, refute or adapt those linkages. This is of importance as the state-building rationale is explicit or implicit in many of the investment models of development agencies [3], including for investments in HRH, and merits better understanding in itself. We briefly examine the concept of state-building and discuss the elements which contribute to it. In this paper, we draw on wider literature on the links between state-building and human, institutional and economic development and then examine evidence for different mechanisms which might link HRH development with state-building, drawing from fragile and conflict-affected settings (FCAS), where the notion of state-building is most applicable. Some challenges and enablers that can support or hinder the HRH elements of these linkages are highlighted. In our concluding sections, we review the evidence for the linkages and contributions of different disciplines and communities to the discourse.

## Methods

### Composition of group

The authorship group was constituted in a participatory way, following an open call made through the LinkedIn Group on Health Systems in Fragile and Conflict-affected States (FCAS) [4] in December 2014. The FCAS LinkedIn group, which was set up in 2004 under the Health System Global aegis, has over 300 members whose profiles suggested theoretical and/or practical expertise and interest in the topic. Out of this appeal, and through personal networks, an authorship group was assembled which included experiences of different FCAS settings and also different disciplinary backgrounds. The group met virtually on several occasions in order to agree on the scope of the article, discuss the research methods and compare preliminary findings. Each group member was given a different area of literature to explore.

### Literature review

We recognized that state-building is an inherently political concept and therefore we chose to explore studies and literature from a wide variety of disciplinary perspectives. Our scoping of the literature explicitly looked for themes in different bodies of literature, including political sciences, economics, history, public administration and public health. The search was not systematic or exhaustive but rather exploratory. Key words used included the following: [health workforce, health workers, human resources for health] AND [conflict, fragile states, post-conflict] AND [state building, legitimacy, equity, gender, peace, stability, intersectoral, public administration, civil service, political settlement, discrimination, neutrality, international aid, development, aid, security, governance].

For the economics literature, the search was conducted on *JSTOR* (Economics and Business journals), *Econpapers*, the *National Bureau of Economic Research* (NBER) and *Centre for Economic Policy Research* (CEPR) web sites and *Econlit*. With the exception of *JSTOR*, the query gave few answers, and the research was extended to articles containing the term “state-building” only. For historical research, the search was conducted on *JSTOR* (history journals), giving 80 results that were all analysed. In addition to this, another research that only included the keywords “state-building” and “human” found 1 245 entries, of which the first 250 were analysed. Most of them proved irrelevant for the present study.

The research done in political science on state-building and related issues is vast. The approach chosen for this study was to start with the reviews carried out by the Governance and Social Development Resource Centre (GSDRC) in three topic guides [5-7]. These guides are mostly aimed at development agencies and review “grey” literature (such as agency-commissioned reports) more than peer-reviewed publications. Based on the references mentioned and a rapid internet search, further studies were included.

For the conflict and fragile states literature, we focused on *Conflict and Health* and *Disasters* journals and from there snowballed into references from papers. Grey literature was obtained from different web sites such as WHO, MSH, MSF, CfBT Education Trust, USAID, DFID, UNSW, World Bank and Humanitarian Exchange. The public administration search was done by starting with some key references and snowballing from their reference sections.

### Conceptual framework

Based on an initial discussion of the literature, a conceptual framework was developed which was used to structure the findings.

### Case reports

In addition to probing the different bodies of literature, three case reports were selected for a more in-depth analysis. The aim of the case reports is to link the themes more clearly with a concrete context and history. Their choice was based on the areas of expertise of group members. It is not a systematic sample, though the cases do represent different scenarios and geographical areas, and the perspectives of the authors are personal.

## Findings

### State-building—a disputed concept

**Figure 1:**
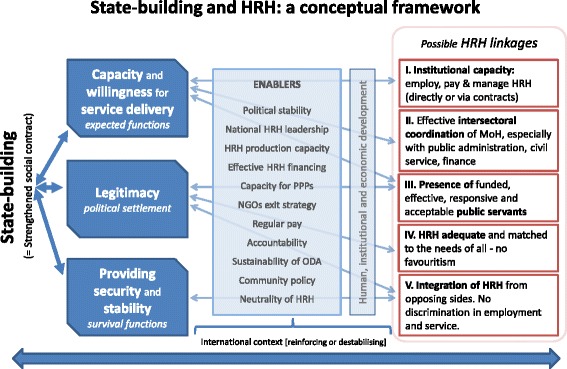
Conceptual framework.

The conceptual framework (Figure 1) starts with state-building as an end-point. It was not a focus to probe the concept of state-building in itself; however, it has to be recognized that this is a disputed concept. Fukuyama [8] defines state-building as the creation of new government institutions and the strengthening of existing ones. He argues that in building states it is important to consider the scope and the strength of the state. The scope of the state refers to the different areas in which the government is able to produce policies and define interventions while its strength is the ability to enforce these policies or to implement these interventions successfully [9]. This is the broad approach used in this paper.

While state-building is often presented as a neutral concept, others have pointed to the inherently political nature of the project and to the fact that building of states can mean reinforcement of oppressive regimes. This includes the role that HRH (the subject of our study) may play in this process. There is a great wealth of historical literature on how colonial medicine and health services, through the medical workforce, contributed to establishing unfair and coercive states [10,11]. Lyons [12] gives a particularly compelling example with the case of “sleeping sickness” prevention in 1900–1940 Congo, which facilitated the building of the colonial state. This example, which echoes other cases around the world, highlights the fact that strengthened human resources in health can also become, through the health system they service, accomplices of the building of oppressive states (to which they bring legitimacy).

Other critics have taken issue not so much with the overall goal of state-building as with its realism, with externally driven, short-term programmes unlikely to create the robust institutions needed to “build” a state [13]. They argue that there is limited evidence of the efficacy of state-building and that indeed it may be counterproductive, undermining the very social legitimacy it is intended to support [14,15]. This point is echoed in empirical work by economists on modern state-building (especially in relationship to aid) that underlines the need for both iterative and localized solutions to institution- and capacity-building [16] and the fact that the “the macroeconomic framework [that aid and donors bring in is] in general much too complex for the available human resources” [17].

However, it should be noted that this critique is aimed at external interventions, and in this paper, we take the concept at its broadest, to include and indeed focus on internally driven processes.

### The “building blocks” of state-building

Our framework identifies three core areas which are hypothesized to contribute to state-building, which are (1) capacity and willingness to provide core services to the population, (2) legitimacy, and (3) providing security and stability. These are broadly supported within the political sciences and economics literature [18]. In particular, Brinkerhoff [19] argues that “governance reconstruction” in failed states and post-conflict societies is articulated around three dimensions:

- Reconstructing legitimacy, i.e. “the acceptance of a governing regime as correct, appropriate and/or right”. Legitimacy does not consist only in participation, inclusion, accountability and contestability (elections) but also refers to delivering services, which links to the “effectiveness” dimension below. The ability to deliver social services is seen to demonstrate government willingness and capacity to respond to citizens’ needs and demands [19].
- Re-establishing security, which includes effective disarmament, demobilization and reintegration (DDR) processes.
- Rebuilding effectiveness, which refers to the delivery of core services (health, education, electricity, water and sanitation, infrastructure, etc.) at the central and sub-national levels. Economic governance is also included here. This also recognized by the Organisation for Economic Co-operation and Development (OECD) [20], which argues that, in turn, the integrity and effectiveness of the civil service will influence the legitimacy of the state. The link between the presence of civil servants (including HRH) and legitimacy of the state therefore runs in both ways, and while the presence of capable civil servants is essential to ensure the functioning of public administration and service provision, their presence is unlikely in the total absence of state legitimacy.

As the OECD argument on the linkage between effectiveness and legitimacy shows, these three dimensions seem necessarily closely linked. This constitutes the base of the “whole-of-government” approach that the OECD and many donors are increasingly adopting in their intervention in post-conflict and fragile settings, integrating defence, diplomatic and development agencies [21-23]. The donors’ literature generally identifies the same [24] or similar three dimensions of state-building; the World Development Report 2011, for example, stresses the ability to provide security, justice and jobs [25], while the Department for International Development (DFID) focuses on political settlement and processes, core functions and expected functions [26].

Other authors (see, for example, [27]) make a slightly different analysis of state-building, differentiating between constitutive and outputs domains. Constitutive domains include political settlement and regime, security, rule of law, administrative governance; while output domains include service delivery (health, education, utilities, etc.), justice system and processes and economic governance. Similarly, Haider [7] makes an interesting distinction between tangible and intangible aspects of state-building. The first group includes actions such as demobilizing soldiers, restoring infrastructure, building institutions and providing services, while the second includes promoting positive state–society and intra-society relations; restoring/generating trust in government, public institutions and among citizens; and fostering socio-political cohesion.

Based on this review of the theoretical literature on state-building, we identify two possible pathways through which the legitimacy of a state may be increased with reference to HRH:

1. Provision of health services (of which HRH is a key element) leading to increased state responsiveness to citizens’ needs, which in turn increases legitimacy in their eyes.
2. Presence of publicly employed HRH (civil servants) leading to the presence of a legitimate and responsive state, which increases trust and legitimacy of the state.

In a sense, these pathways could be seen as pertaining, respectively, to the tangible and the intangible aspects of state-building described above.

### State-building and human, institutional and economic development

HRH development clearly intersects with state-building via a wider web of human, institutional and economic development. State-building *per se* is a relatively recent concern for economists, who have, however, been interested in the role of institutions and human capital in economic growth and development for a longer time [28]. There is a well-established literature on the role of what economists call human capital, often narrowly understood as (formal/school) education, in generating economic growth through innovation and gains in productivity [29-31]. Economic surplus and growth are, in turn, perceived as fundamental elements for the emergence of states, which are deemed as resting on the protection of productive economic interests (especially security) in exchange for taxation [32]. Contrary to post-war growth theories [33], recent neo-classical growth theories argue that human rather than physical capital is the lead engine of growth [34].

However, institutional economics and especially the influential work of Acemoglu and Robinson [35] tend not to put human capital at the centre of (state) development. “Good institutions”, which equates to inclusive ones, matter much more than human capital and are also not necessarily the product of higher human capital, argue for instance Acemoglu et al. in [36]. Similarly, economic historians such as Khan [37] challenge the mainstream understanding of human capital, showing that in the building of Western European states *ad hoc* “commonplace skills and entrepreneurial abilities to resolve perceived problems” matter much more than formal and deep technical knowledge.

Most theoretical and applied economists nonetheless agree on the idea that support of human capital development is one of the state’s key functions [38,39]. They assume that strengthened human capital has positive impacts on markets and growth, although this impact may not always be visible in the short run. The latter, as well as “weak constraints on the ruling group” and “political instability”, lead theorists [38,40] to argue that weak states have very little incentives for investing in human capital and may develop human resources (and other investments) “outside of state structures”.

On their side, historians advance the nature of the political and professional elite as an explanatory factor for state-building. Looking at the cases of Brazil and Chile, de Carvalho [41] shows that a common ethos among the elite as well as social and ideological homogeneity favoured the consolidation of effective political power and the building of these states outside of Spanish and Portuguese rule. However, this homogeneity can quickly lead to a dead end as power is not being redistributed. This is also the point of Waldner [42], who looks at successful development stories in the 20th century and identifies broad coalitions of the existing human resources as necessary for sustainable development. The importance of the civil servants’ ethos and ideology in the building of the state is highlighted by many other authors, including Anderson [43] who works on early modern Germany. It is more generally reflected in the distinction between *nation*- and *state*-building (the latter is stripped of the ideological dimension).

This leads to the case of the “illiberal state-building” of Angola, where the elite is invoking, among others, the lack of human resources to justify a different state-building “in defiance of liberal peace precepts on civil liberties, the rule of law, the expansion of economic freedoms and poverty alleviation” [44]. De Oliveira, in company with most historians and economists, is doubtful of the real commitment of predatory elites to human resource development.

The connection between the civil service and the citizens is identified by historians, such as Olowu [45], as a key factor for effective state-building as it should enable adequate goods, services and efficient markets. Looking at the argument the other way round, he highlights that “the disconnection between the state and society is one of the fundamental causes of the crisis of the African state and its civil service systems”.

## Linkages between HRH and the state-building nodes

### Institutional capacity for health workforce governance

In FCAS, governments struggle to mobilize, manage and distribute health resources. Often, health care is delivered either through public health services or by contracting out to third parties (e.g. NGOs). For example, in post-conflict Sierra Leone, health services were provided through public facilities [46], while in Afghanistan [47] and initially in Timor-Leste [48] NGOs were contracted to provide health services to the population (see Tables 1 and 2). Sometimes, particularly during the early stages f post-conflict, NGOs deliver services in areas where security is still volatile while government covers urban and more secure areas. This is, for example, the case of South Sudan where NGOs were reported to deliver 84% of health care services, while government took responsibility for the other 16% [49].

**Table 1:** **Afghanistan case report**

**Theme**	**Findings**
Background	In 1978, the former Soviet Union military invaded Afghanistan, leading to chronic conflict, insecurity and instability in Afghanistan. The communist regime remained in power until 1992, and during the 13 years of its ruling, it contributed little to the welfare of the people. After taking power in 1992, a coalition of Mujahedeen factions brought Afghanistan into a new time of conflict, civil war and inter-Mujahedeen fighting. The Taliban ruled the country from 1996 to November 2001. The Taliban showed little interest in the health sector [73]. In December 2001, the Taliban regime collapsed and a new democratic government was established. In 2002, the level of health services was shocking. Lack of a policy framework, inequalities in health service provision across the country, low capacity of public and private sectors, differences in the quality of the services, the absence of infrastructure, lack of coordination and shortage of health human resources were some of the main challenges [74].
Institutional capacity	After the establishment of the new government, the *institutional capacity* of the Ministry of Public Health (MoPH) at the central level has been strengthened with the provision of training programmes and the hiring of a number of local consultants. Regulatory documents and guidelines to support the hiring and management of HR have been put in place in collaboration with the Civil Service Commission. Despite these efforts at the central level, at the decentralized level, the state capacity to provide services is still weak. Scholars working on public administration and civil service (beyond the health sector) in Afghanistan have highlighted the gap between the formal and informal institutions. The limited reach that the *de jure* state has in the provinces leaves room for a *de facto* authority structure of warlords, with commanders filling in the state functions and weakening its legitimacy [75].
Intersectoral coordination	Intersectoral collaboration has been reinforced by increasing coordination and dividing tasks between different institutions within the public administration so that the Civil Service Commission hires top grade officers (general directors and directors), while the MoPH is responsible for hiring all other officers. However, the institutional capacity is not at optimal level. Moreover, the financial capacity of the government to pay its officials remains limited. Most of the staff in key positions of the MoPH, while officially employed by the government, receive a salary or a salary supplementation from external organizations and development projects. Although work has been done to attempt to align and harmonize pay, disparities in remuneration still exist, which are a cause of demotivation for health workers (HWs) [76,77].
Adequacy and coverage of HRH	In contrast to other services provided by the public administration, health service delivery at the decentralized level has been contracted out to NGOs [60]. In terms of the presence of funded, effective and responsive HRH, NGOs hire health workers directly. In 2003, the MoPH developed a national salary policy to standardize HWs’ remuneration across the country and compensate particularly female HWs for assuming posts in rural and underserved areas [78]. The remoter the HWs, the higher the salaries they receive. However, due to the lack of health care workers, especially of higher cadres, in rural areas and the high security risks, disparity in health provision and inadequate HRH compared to the population needs is noticeable. The most recent *HRH Strategy* points to gender imbalances as well as disparities in urban/rural distribution of HWs—for example, there are 16.7 public health workers per 10 000 (including unqualified support staff) in rural areas, compared with 36 per 10 000 in urban areas [76]. These issues may contribute to the problems that characterize the health system and the weak public health care provision. Evidence at household level suggests limited utilization of (NGO-provided) public health services, perceptions that these offer inferior quality and a preference for private providers [79].
Integration fo HRH	While official regulations do not allow for discrimination in provision of employment, in practice, it is difficult to ensure a non-discriminatory environment and transparency of hiring practices. Political and tribal pressures exist, and favouritism and nepotism are common when hiring new staff, particularly in key positions, also at the central level [77]. It has been noted that the preferential support of donors for discrete health programmes and the establishment of parallel management systems outside the MoPH, rather than broader institutional support to the MoPH, including to anti-corruption and transparency programmes, may hamper the progress made on these issues, which are essential for the improvement of the public health sector [77].
	Reinforcement of the public civil service for the provision of health care services seems to be following two tracks. At the central level, the presence of a cadre of professionals that are well-trained and relatively well-paid (with external salary supplements) seems to be playing a role in contributing to the state-building process (despite some persistent difficulties). At the local/decentralized level, however, improvements in the availability, distribution and adequacy of HRH seems to be hampered by insecurity so that the strengthening of HRH (and health service provision) appears to rest on state-building rather than contributing to it.

**Table 2:** **Timor-Leste case report**

**Theme**	**Findings**
Background	Timor-Leste achieved its independence in 2002 after 450 years of Portuguese colonization and 24 of Indonesian occupation. Following the vote for Independence in August 1999, an Australian-led multinational force was deployed to stop the violence and destruction triggered by the results [80]. A UN Transitional Administration (UNTAET) was established in October 1999. The country’s health system faced serious problems due to destruction of infrastructure and severe health workforce deficiencies as skilled health workers and managers fled the country [48,53]. NGOs were the main health service providers during the emergency period from 1999 to 2001. A Trust Fund for East Timor (TFET) administered by the World Bank was created in early 2000 [53]. Timor-Leste faced a relapse of violence in 2006, and a UN mission (UNMIT) was deployed. In preparation for its withdrawal in 2012, the country developed a transition plan in 2011 to move from peacekeeping “*…to the new phase of state building*” [81].
Institutional capacity	Weak institutional capacity was part of the legacy left by Indonesia after withdrawal in 1999. During the Indonesian occupation, most middle and top managerial positions in the government, including the health sector, were held by non-Timorese Indonesians [82]. Around 7 000 civil servants fled the country after Indonesia’s withdrawal [83]. In the health sector, destruction of health facilities and institutions, including destruction of records, left severely weakened institutions. UNTAET and the first National Government had as a priority to develop individual and institutional capacity. In the health sector, training of district managers and senior officers working at MoH level was prioritized and undertaken as early as 2001 and 2002 [51]. This allowed, for instance, for the assumption of responsibility over district health management by the government. However, there was a sense of scepticism about this decision among NGOs and development partners who thought there was not enough capacity built yet to ensure an efficient health service provision [48] which speaks about the limited international legitimacy of the emerging government. Soon after the initial emergency phase, strengthening educational institutions for health professions was prioritized. Establishment of a Faculty of Medicine in 2005 and schools of Nursing and Midwifery in 2008 allowed for local production of these key cadres. This contributed to a relatively more sustainable workforce than in other small countries in the region which still depend on international recruitment and on sending students abroad.
Intersectoral coordination	The Timor-Leste Ministry of Health’s vision implies a broad definition of health which involves social determinants of health [84]. This approach requires intersectoral collaboration. The Strategic Development Plan 2011–2030 recognizes that in order to address health problems an intersectoral approach is required and that coordination with other sectors such as agriculture, environment or infrastructures is paramount [85]. However, the implementation remains a challenge due to HR and institutional capacity limitations [86].
Adequacy and coverage of HRH	Deployment of skilled health professionals to remote areas as part of the government’s policy to staff each facility with one doctor, two nurses and two midwives in every village is currently ongoing. However, while deployment of physicians is already achieving 76% of the target (335 of 442 TL’s villages), appointment of nurses and midwives is proving more difficult mainly due to the more limited production of these professionals^a^.
	MNCH indicators in Timor-Leste are still poor. Access to MNCH service in remote areas is limited mainly due to shortage of adequate HRH. In order to address this issue, Timor-Leste is currently supporting nurses with rural backgrounds to undertake training in midwifery to ensure their deployment and retention in these remote locations [87]. During the pre-Independence period, assistance during delivery in Timor-Leste was usually provided by traditional birth attendants (TBAs), partially due to the mistrust of the population on the Indonesian health services. Ribeiro Sarmento [88] found that the integration of TBAs in the public health workforce as family health promoters contributes to increase access to these essential services to population living in remote areas hence contributing to increase equity in health service delivery.
	Only 31 doctors remained in Timor-Leste after the withdrawal of Indonesia [58]. The government of Timor-Leste signed a bilateral agreement with Cuba in April 2004 to deploy between 150 and 200 doctors to provide clinical services and to train 1 000 Timorese doctors [89]. As a result, 838 medical doctors graduated between 2010 and 2014 and are now being deployed across the country including remote areas [90].
Presence of funded, effective and responsive public servants and CHWs following public goals	Weak institutional capacity within the transitional administration was reflected in the slow pace of the process of recruitment of civil servants in 2001, which is reported to have undermined the credibility of the newly established Civil Service [53]. However, initiatives like the reorientation and integration of TBAs within the national workforce and the scaling up of the midwifery workforce mentioned above are contributing to improve the availability of services provided by these key professionals.
Integration of HRH: the role of HRH in the 2006 political instability	After some years of peace, political instability caused widespread communal violence in 2006, leading to the displacement of approximately 150 000 people. The conflict deepened the division in the Timorese community between “East—*Lorosa’e*” and “West—*Loromonu*”. Timorese health workers belonging to both sides of this divide played a commendable role avoiding being dragged into this division and continuing to work maintaining their neutrality and impartiality and providing health care to people from East and West without discrimination. Cuban doctors and NGO staff played also an important role. Strong leadership by the Minister of Health, communication and effective coordination are among the factors identified that kept staff morale to continue working in such an unstable environment [90]. This contributed to promote a sense of resilience among the people living in IDP camps and the general population who was able to access health services during the crisis without disruption. This is likely to have contributed to increase the government’s legitimacy. Dr. Araujo, former Minister of Health and regarded as the leader of the health system rehabilitation, has been recently sworn in as the 5th Prime Minister. This shows the key role that health professionals, thanks to their social legitimacy, can play not only in reconstructing health systems after conflict but also in assuming leadership roles and contributing to national reconstruction and state-building.

^a^Personal communication with the Director of the Human Resource Department of the Ministry of Health Timor-Leste.

While delivering services through third parties is suggested to undermine a government’s legitimacy by some authors [50], others maintain that whether there is a loss of legitimacy or not depends on the role that the state plays on coordinating the work of NGOs or other private providers. In Afghanistan, the MoPH developed national health workforce policies including HR management systems. However, as capacity for health service delivery is still limited, the MoPH signed contracts with NGOs allocated to provinces, and NGOs are bound by a formal contract to deliver a Basic Package of Health Services (BPHS) at the provincial level [51]. This shows some control over the NGOs’ work by the MoPH [2]. A similar strategy was used in Cambodia [52]. In both cases, the state retains its stewardship role and is seen as responsible for the provision of services; therefore, its legitimacy should not be affected. In Timor-Leste, the European Union shifted from funding NGOs directly to funding them via the government, allowing the Ministry of Health (MoH) to sign contracts with NGOs detailing a list of services that they had to deliver at the district level [53]. As security improved and the transitional government was in place, in 2001, the government took over the management of health services and staff from NGOs, while requesting NGOs to continue providing technical and logistical support. How much each different strategy (e.g. direct public provision or commissioning/contracting out) contributes to improve the legitimacy of the emerging government is again difficult to ascertain, and there is no empirical evidence of the linkage of the type of service provision with state legitimacy.

### Equitable availability of HRH

The training and deployment of community health workers (CHWs) is often chosen as a means to ensure service provision in remote areas, which in turn is hypothesized to show willingness of the emerging government to provide services for all, hence contributing to state-building. It is reported that the introduction of teams of CHWs increased access to antiretroviral therapy in remote areas of Mozambique [54]. Similarly, in Afghanistan, CHWs—more than half of whom are women [55]—arguably contributed to strengthen the health system by expanding accessibility to TB and maternal, neonatal and child health (MNCH) services [56]. In Pakistan, the Lady Health Workers programme also reportedly improved access to and utilization of MNCH services [57]. The fact that these CHWs contributed to increased acceptability of health services may have promoted the image of the government as willing to provide services to their constituencies. However, in the case of Pakistan, the popularity of the programme was attributed to the support received from the Prime Minister, who strongly promoted this strategy, which demonstrates the possible two-way links between politics and HRH investments. Whether CHWs are integrated in the national workforce with a specific role or contracted by NGOs and therefore working more independently may influence their impact on state-building.

During early post-conflict interventions, donors and governments often need to show quick results, and priorities may follow externally driven political objectives rather than health needs. Priority is then given to the rehabilitation of the more visible physical infrastructures, such as tertiary hospitals, and peripheral structures that serve people who have less access to health care and often greater health needs are neglected. Similarly, in HRH, priority for rehabilitation of the different cadres may not be guided by equity concerns: the medical workforce is sometimes prioritized above the nursing workforce, despite the fact that nurses are the main providers of basic services, particularly in more deprived areas where the population most in need lives [58]. For example, in Cambodia, nurses and midwives trained before the war were retrained as doctors. This took the experienced HWs of these cadres away from their original professions, causing an over-production of doctors and medical assistants. Moreover, pressures were in place to abandon training of assistant-level nurses and midwives in favour of training diploma nurses, who often then ended up working in the private sector^a^.

Periods of post-conflict are often said to open windows of opportunity to improve the efficiency of existing institutions as resistance from politicians and civil servants is often less intense than during periods of stability. In Timor-Leste, an inflated nursing workforce during the Indonesian times was reduced from more than 3 000 to 1 400 after the conflict [53]. In Mozambique, the MoH removed around 2 000 ghost workers from the payroll in the early 2000s [55]. Pavignani and Colombo suggest that the reconstruction process represents a unique opportunity to improve equity in human resources within the health system [59]. The precise timing of this window of opportunity can vary; a study of HRH policy in Sierra Leone found that the window for reform opened not in the immediate post-conflict period, when capacity and authority were still limited, but after some years, when government and donor commitment coalesced around reforms linked to the Free Health Care Initiative [46].

Despite these examples, it has been shown, however, that in many countries affected by conflict the government is often not able to provide health services. Therefore, networks of formal and informal health workers with different backgrounds and skills emerge to provide different services demanded by the population [60], including training [55].

### HRH reintegration and inclusiveness

The reintegration of “civil service personnel” of opposing factions may play a relatively important role in reconciliation and peace-building. In a review of the DDR process of Ethiopia, Colletta et al. [61] find that, among all the activities carried out to reintegrate ex-fighters, only HWs have a special status (1 400 ex-combatant health workers were employed by the MoH at the end of the conflict). In Sierra Leone, there are descriptions of how a polio campaign and EPI vaccinations became an entry point for the negotiations with the Revolutionary United Front (RUF) rebels towards the end of the conflict in 2000–2001 for the delivery of health services behind rebel lines. In terms of HRH, “training and implementation of the exercise had a 60% RUF, 40% MOHS personnel” [62] and it was key to involve local (qualified and unqualified) personnel from the communities. The “combat medics” who were providing services for the RUF and for the population behind rebels’ lines had little or no formal qualification. Initially, they were included in the campaigns only for the social mobilization aspects. However, the “combat medics” themselves later demanded to be included as vaccinators and were eventually trained by the District Health Management Team (DHMT). Later on, they were mostly employed to carry out vaccinations in the border areas between Sierra Leone and Guinea which were still unsecure. The collaboration between DHMT/government HWs and RUF combat medics is often stressed as one of the keys of the success of the campaign and the subsequent beginning of MoH provision of health services in the Kailahun district^b^. At least in the informant’s experience, deployment of HWs hired and paid by the MoH in all districts, reintegration and training of “HWs” (including the unqualified ones) formerly working with the rebels and local recruitment all played a key role in reinforcing and entrenching the health systems in rebel-affected areas.

Balladelli et al. [63] suggest that investment in addressing integration of military health workers requires substantial political and financial efforts but that it is greatly justified. In Angola, more than 1 500 UNITA health workers were reintegrated in the national health service workforce after the conflict. The positive experience was then extended to around 7 500 civilian health workers who worked in areas dominated by UNITA during the conflict [63]. In Mozambique, reintegration of HRHs who worked in areas dominated by RENAMO within the new health system helped in dissipating tensions and contributed to the “good image” of the emerging government [59]. However, there is no empirical evidence about the causal relation or the contribution of the reintegration on improving the legitimacy of the new government or its overall contribution to state-building. It is also to be noted that 20 years after the conflict in Mozambique the divide between the government and the rebels is still wide, and the civil service remains loyal to the ruling party. There are other examples where this integration did not take place. The ratio of Hutu senior government officials per Hutu population in the Rwandan civil service 15 years after the conflict was 1 per 500 000 Hutus while the proportion of Tutsi senior public servants was 1 per 70 000 Tutsis [64].

Although humanitarian workers are important actors in post-conflict settings, it is hard to measure the impact of their interventions on state-building in fragile areas. The role of NGOs often goes beyond the mere delivery of services; it also includes other elements such as the witnessing of human rights abuses by health professionals [65]. There are many positive examples of these interventions, such as humanitarian ceasefires for immunization campaigns in El Salvador or transport of medical supplies in Uganda and Sudan [66]. The provision of health care can be a common ground for collaboration between health professionals from opponent parties, which in turn can contribute to community reconciliation. Continuity in the delivery of health services during relapses of violence after conflict may be perceived positively by a population already traumatized by the recent conflict. In Timor-Leste, the behaviour of the health workforce during the relapse of violence in 2006, which continued providing services despite the turmoil, was reported to have provided a sense of resilience and continuity within the chaos [67]. This particular case demonstrated that health workers were impartial during the conflict and were able to fulfil their professional ethics in times of instability, but this may be an exceptional case rather than the norm.

### Enablers and challenges

In the framework proposed, we also identified a series of enabling factors and potential challenges which could strengthen or hinder the linkages between HRH and state-building processes, and we explored the related literature, although not systematically. Many of the concerns (indeed, more challenges than enablers emerge) come from donor-oriented literature. It focuses on lessons for external partners, including the long-term approach which is needed to support successful bureaucratic reforms, a time span which does not fit with the length of typical projects [68]. The need to trade-off short-term goals (re-establishing service delivery) against long-term ones (capacity-building) is also highlighted by a number of authors, such as Fukuyama [8]. Timor-Leste, however, in reconstructing the civil service after the conflict, prioritized capacity-building on general and financial management as a means to strengthen policy implementation. In the early stages of the health sector rehabilitation, priority was given to train mid-level managers (e.g. DHMTs) in order to ensure an efficient management of the newly recruited civil service [53]. As a result, responsibility for health services management was swiftly transferred from international NGOs to government at a relatively early stage in the reconstruction process with no impact on the positive trend of health service delivery output indicators [48]. Another important lesson is that reforming human resource management and development practices is an inherently political process—a fact that donors ignore at their peril [68]. The need for leadership development, adequate remuneration [69], harmonization with wider public administration reforms and developing capacity to manage relationships with non-state actors are also emphasized.

## Discussion

We have found that there is a substantial body of evidence on state-building and its constituent parts, largely drawn from political and historical sources. From a historical perspective, the process is often associated with an accumulation of capital, centralization and control over the means of coercion and the enhanced power of rulers to access these resources by appearing to be legitimate [15]. When the term is used by development agencies, it clearly has different connotations, being used in a politically neutral and more sanitized manner, to mean support to national institutional development. There is some critique of the concept of externally engineered state-building, both in development and economics literature. In this paper, we have considered the relationship, theoretical and empirical, between human resources for health development and state-building, understood as the (largely internally driven) processes whereby the capacity, legitimacy and ability to provide security of a state are enhanced.

A strand of the literature has analysed the relationship between health sector development or health system strengthening as a whole and state-building focused on fragile and conflict-affected countries. Kruk et al. [2], for example, identified strengthening state institutions and legitimacy as the basis for establishing a resilient state after conflict, while the legitimacy of the new government in Mozambique after the conflict is reported to have been strengthened by its focus on the rehabilitation of primary health care services in rural areas [59]. However, much of the literature is theoretical or normative, setting out expected and hoped-for relationships. There is very limited empirical evidence, which is not surprising, given the sometimes intangible nature and difficulty of assessment of state- and institution-building. Where evidence exists, as in Mozambique, the causality is unclear and can be assumed to run both ways—greater legitimacy allows the government to develop primary health care (PHC) services as much as better PHC services contribute to legitimacy.

There has been no review to date, to our knowledge, of the specific contributions of human resources for health to state-building. We started by outlining some possible linkages. One is through institutional capacity development to employ and manage staff, directly or indirectly through contracts with third parties. This is discussed in public administration, political and development literature. Within public administration, perhaps unsurprisingly, the restoration and strengthening of the civil service is seen as the backbone of state-building—supporting all of the core nodal areas, such as delivery of services, increased legitimacy, intersectoral coordination and reintegration of staff. The focus of the literature is on the measures needed, and the relationship with state-building is largely (and some might say reasonably) assumed.

Managing relationships with non-state actors has provoked a large and ideologically divided literature. On one side, state-building evidently focuses on the capacity of the public bodies to either deliver services or to manage their delivery effectively. In FCAS, however, the capacity to do either is often lacking, and supporting service delivery by third parties, often international NGOs, has been contentious in terms of its long-term legacy. In some cases, such as Afghanistan, staff are not hired directly by government, and in others, such as Timor-Leste, NGOs initially hired staff directly but soon switched to working alongside government hired and managed personnel. The latter model is seen as having developed government capacity, but the familiar chicken-and-egg problem arises. More fundamentally, some oppose the statist focus and highlight the contributions of a wide range of formal and informal providers to the resilience of the health care market, particularly in FCAS. State-building in this context is seen as ignoring or potentially threatening the often important role these actors play.

There is a growing literature within health and conflict writings about the importance of supporting adequately trained, supported, distributed and managed staff in FCAS and how this may be achieved, but the links with the state-building are not explicit. Economic literature, however, identifies some elements within “human capital development” which contribute to economic development, which in turn is affected by and affects state development.

More clearly connected are the cases of reintegration of staff from different factions, post-conflict, which not only establish services which are nationwide but also send clear messages of reconciliation and non-discrimination. Positive anecdotal evidence comes from a range of countries, including Sierra Leone, Angola and Mozambique, though there are also examples of countries where this has been hard to achieve (such as in some of the Balkan states post-conflict).

Much of the literature identified is aimed at development agencies and has a pragmatic orientation towards lessons learned, without taking on board some of the broader political and historical aspects, which come from the wider (non-commissioned) studies. The latter literature, of whatever disciplinary background, has a more sceptical approach, particularly to externally driven state-building. The risk of linking success indicators for projects to something as intangible and hard to measure as “increased legitimacy” and other state-building “outputs” is rightly highlighted [70].

More plausible in the long run is the contribution of HRH to the development of professional associations and public service institutions, which ultimately build capacity, legitimacy and security, as well as contributing to the economic growth which underlies these. Historical analysis is best suited to unpicking these relationships, though always open to (re)interpretation.

None of the relationships are simple, mechanical, one-directional or static, however. As highlighted in the framework, history demonstrates virtuous as well as vicious circles. Power and institutions can be used oppressively, corruptly and to deepen inequalities. *De jure* and *de facto* relationships differ, and local and national systems can be very divergent, as illustrated by the Afghan case report. As medical staff often represent an elite group within their societies, with greater access through their training to political office [71], they may have an influence (positive or negative) beyond other public servants. There are also trade-offs to be managed. Looking at the case of state-building in the Solomon islands from a long-run perspective, Dinnen [72] finds that “the capacity-building paradox is that the more substantial the intervention is in terms of external resources and personnel, the greater the risk that it ends up ‘sucking out’ local capacity rather than building it”. In his view, both capacity-building (HR strengthening) and service delivery are essential for state-building, but they are often antagonistic, and there is, ultimately, a trade-off between quickly restoring service delivery on the one hand and building capacities on the other.

The literature (especially studies which are not written for donor agencies) suggests that we should be cautious about intervening externally in order to “build states” through HRH development as well as well as other channels. The complexity of local dynamics is such that the risks of unintended negative consequences are high. Targeting more tangible outputs, such as improved service coverage, is advisable. Even here there are risks and trade-offs, of course. The example of the PBF system in Burundi (see Table 3) highlights that there is no magic bullet (in this case, the issue of sustainability rapidly arise).

**Table 3:** **Burundi case report**

**Theme**	**Findings**
Background	The post-colonial history of Burundi is affected by long periods of autocratic rule (1962–1992), mass killings (1972, 1988, 1993) and a protracted civil war that started in 1993 and only definitely ceased in 2008. Democracy, the outcome of the liberal peace that gradually started with the Arusha Peace agreements in 2000, is still fragile, and the recent years have seen continuous political violence and the control of the political and economic power by a small group of people coming from the ranks of the former main rebel movement CNDD-FDD. Burundi remains a very obvious case of a FCAS. The country ranks at the very bottom of most rankings on health, human development and governance. The war left the health sector in ruins [91]: in the early 2000s, as peace was returning, the WHO estimated that the country had only 2 nurses per 10 000 inhabitants. The last available figure from 2009 is 19 nurses per 10 000 inhabitants.
Institutional capacity	As a 2011 MoH report points out, information on the management of the health workforce at district and health facility levels is still lacking. The WHO-sponsored National Observatory of Human Resources set up in 2012 may help improve the situation by gathering intelligence on HRH and strengthening institutional capacity to manage them. However, in general, public servants’ positions and tasks within the MoH and at the peripheral level are still often not clearly defined in job descriptions [92]. Laws have been passed (notably the 2010 HRH Development Policy) and frameworks have been designed to improve the management of human resources, but in the field, difficulties remain [93]. In 2006, at the same time when user fees were removed for pregnant mothers and children under five, international NGOs started implementing performance-based financing (PBF) mechanisms in order to respond to the challenges of responsiveness and effectiveness of the public servants. PBF has also boosted the regularity of nurses’ payment and their mean income, which now approximates US$ 350–400/month (for a qualified nurse). Yet, PBF has not tackled the issue of the actual salaries remaining low. Overall, PBF and international aid may have augmented the institutional capacity of the MoH, hence possibly contributing to improving service delivery, but have also introduced a degree of complexity that may not be manageable without aid support. Official documents show that the MoH relies heavily on PBF to sort out human resource issues, ranging from incentivization and payment to general management [93].
Effective intersectoral coordination	Until 2010, there existed a Ministry of HIV/AIDS alongside with the Ministry of Health—and the lack of coordination between the two ministers was notorious. The divide of ministries between political parties and “ethnic” groups, with the Ministry of Health not necessarily always falling in the camp of the main political party, also contributed to hampering coordination until around 2010 when the CNDD-FDD established a firmer control over the MoH. Formal mechanisms of coordination remain primarily aid-led. They also suffer from the reluctance of the health sector to collaborate with other sectors that did not move as fast as it did after the war. Indeed, the MoH had a clear advantage over other ministries as (1) it did not face the same challenges of reintegration of part of the workforce as other sectors (see below) and (2) could count on a well-identified workforce whose work was not very different from past regimes. In the recent years, the presidency has established a stronger grip on health issues, but often, decisions are taken without consultation with or agreement of the MoH staff. A very clear example is the introduction of a new insurance scheme in 2013 that many in the MoH viewed as badly designed but was forced by the presidency. The coordination of the different actors, including non-state, involved in HRH management still remains a weakness of the health system [93] and maintains Burundi as an aid-dependant state.
Presence of funded, effective and responsive public servants and CHWs following public goals	Although until recently the Community Health Workers have been largely left out of the PBF scheme [94], PBF has provided a new source of revenue to the medical staff and has possibly increased their responsiveness to the population needs in terms of maternal and child health care [95,96]. The definition of (paid) indicators has also provided clear incentives for the public servants to align with the priorities defined by the MoH. As long as the PBF functions and leads to (even small) improvements in service delivery, it could reinforce the state, but the risk is that this elaborate scheme, which still rests on international money and support [96], eventually crumbles. As of 2014, less than 1% of the MoH staff had had training on human resources management, and the lack of clear terms of reference for positions may put at risk the good accomplishment of public goals
Adequacy and coverage of HRH	This is perhaps the area in which most progress has been made and where the linkage with state-building is the most obvious, although the causality is probably going both ways. The Tutsi autocratic rule and the 1972 mass killings [97], which targeted educated Hutus, led to a clear imbalance in human resources in health (especially at the highest levels of qualification). The discrepancy in service provision has been well-documented, with the province of Bururi, home province of the dictators, being clearly favoured [98]. Post-conflict strategies for human resources in health did try to balance this out, and the change of political power (to the hands of Hutu northerners) also changed the geographical focus of patrimonial flows. The imbalance still partly remains, though, with most resources per inhabitant still concentrated in the west of the country, but it is probably less than before [99]. In the long run, it is, however, unclear whether the true beneficiary of this change is the Burundian state or the ruling party (or both). A recent report on health facilities [99] finds that human resources are still too centralized.
Integration of HRH	During the civil war, most of the health facilities remained, officially, under MoH control, and the state was, with support from international aid, the main provider of health services. At the local level, the post-conflict integration of human resources was much less of a problem in health than in other sectors. At the central level, the ruling party eventually took control of the MoH. There is recent anecdotal evidence of a ruling party-induced politicization of HRH down to the level of health centres’ chief nurses (with chief nurses being asked to join to the party), which may have unclear effects on state-building. At the same time, the wider opening of medical training has certainly contributed to creating a medical workforce that better reflects and integrates the political, “ethnic” and social cleavages of Burundian society. There was only 1 private paramedical school in 2007 after the war, and 4 years later, there are 13. However, some reports and official documents have seriously questioned the quality and adequacy of the training provided by the newly created schools of nursing and medicine [92]. The quality control of the sector tends to be loose.
International context	Burundi has benefited from massive international aid, which still constitutes over half of its planned budget (43% in the 2010–2015 PNDS). As in other countries, humanitarian aid and the early phases of development aid took a toll on the few existing human resources (that were diverted from the public sectors to aid organizations). The phenomenon has not stopped with the country officially coming out of the humanitarian phase, although, fortunately, the total number of nurses and doctors has increased. As Dinnen [72] noted in a different case, the positive aspect of international involvement is an improvement of service delivery, but it comes at the cost of a protracted dependency to aid which may be detrimental to state-building. In the past years, autocratic decisions of the government, political intimidation and abuse of human rights have pushed donors to withhold part of their support, putting the country on the verge of bankruptcy and triggering more instability [100].

Ultimately, health, health care and health care workers are all subject to political considerations. The literature on reintegration of staff from different factions, on disease control as a “neutral entry point” and on health as a “bridge to peace” all highlight the fact that health can be used to promote stability and reconciliation but can also be manipulated or “weaponized”. The local political economy is all-important. The Burundi case report, for example, illustrates that increasing numbers of health workers may be easier (in the local political economy) than reorganizing the workforce and dealing with a top-heavy bureaucracy, while party control over a ministry may be key to its influence.

This paper has a number of important limitations. It is based on an exploratory review of the literature only and may have missed some important studies. In addition, the case reports are limited and do not represent the full range of experiences. Finally, we selected a number of disciplinary areas to investigate, which are not exhaustive. Given that the topic cuts across a number of boundaries, the search could have been almost endless. However, on the positive side, the attempt to draw from different bodies of literature has been very illuminating. The discourses clearly vary, and the bodies of literature focus on different parts of the conceptual framework. While the literature on HRH and health systems focuses on pragmatic issues and makes limited assumptions about wider impacts, public administration is more normatively aligned with institution-building and the “middle zone” of the framework, as is economics, with its probing of state–economy relations and underlying drivers. Political sciences naturally focus on the left hand side of the conceptual framework—on conflict, power and states, though with a less naive view of how they are built, compared to some of the development literature. Adding the longer term perspective of history also helps to understand patterns which are not obvious to more short-term studies, although these patterns are still subject to interpretative differences.

## Conclusion

This paper, based on an exploratory literature review and the experience of the author group, has developed a conceptual framework for understanding how human resources for health could contribute to state-building. It has found that the empirical evidence for most of the linkages is not strong, which is not surprising, given the complexity of the relationships and the intangible nature of some of the elements, as well as the research methods which would be involved in attributing causality. Nevertheless, there is plausibility to some of the posited relationships, especially between development of health cadres and a strengthened public administration, which in the long run underlies a number of state-building features, including the capacity to deliver services, legitimacy and provision of stability and security. The reintegration of factional health staff post-conflict is also plausibly linked to reconciliation and peace-building, though clearly this has to be accompanied by other measures which send out complementary signals to the population. The role of medical staff as part of national elites may also be important and merits further study.

The paper draws from a range of literatures and illustrates the advantage (and perhaps also the difficulty) of generating interdisciplinary insights. The concept of state-building itself is highly contested, with a rich vein of scepticism about the wisdom or feasibility of this as an external project. While recognizing the inherently political nature of these processes, systems and sub-systems, it remains the case that state-building does occur over time, driven by a combination of internal and external forces, and that understanding the role played in it by the health system and health staff, particularly after conflicts and in fragile settings, is an area worth further investigation. This review and framework aim to contribute to that debate.

## Endnotes

^a^Personal communication with Joyce Smith, 2015.

^b^Personal communication with Dr. Sas Karbo, MOHS Sierra Leone, 2013.
